# Effectiveness of Tuina Therapy for Patients With Chronic Fatigue Syndrome: Protocol for a Multicenter, Randomized, Open-Label, Assessor-Blinded, Parallel-Group Clinical Trial

**DOI:** 10.2196/94022

**Published:** 2026-09-01

**Authors:** Jun Ren, Shoujian Wang, Sitong Fang, Shanda Xu, Chaoqun Xie, Ming Jin, Xingyu Zhou, Wu Li, Yan Zhao, Xingquan Wu, Cheng Wang, Yanguo Wang, Zhichao Gong, Jing Zhou, Junjie Yao, Xue Bai, Yu Wang, Min Fang, Lingjun Kong

**Affiliations:** 1 Department of Tuina, Shuguang Hospital, Shanghai University of Traditional Chinese Medicine, Zhangheng Road, Shanghai, 201203, China, 86 18918134827; 2 Acupuncture and Tuina Rehabilitation Center, Hunan University of Chinese Medicine Second Affiliated Hospital, Changsha, China; 3 Rehabilitation Medicine Center /Tuina Department, Hubei Provincial Hospital of Traditional Chinese Medicine, Wuhan, China; 4 Department of Tuina, The Affiliated Hospital to Changchun University of Chinese Medicine, Changchun, 130021, China; 5 Department of Tuina, Affiliated Traditional Chinese Medicine Hospital of Xinjiang Medical University, Urumqi, China; 6 Department of Rehabilitation, Second Affiliated Hospital of Tianjin University of Traditional Chinese Medicine, No. 69, Zengchan Road, Hebei District, Tianjin, 300250, China

**Keywords:** chronic fatigue syndrome, Tuina therapy, fatigue, randomized controlled trial, protocol

## Abstract

**Background:**

Chronic fatigue syndrome (CFS), also known as myalgic encephalomyelitis (ME), is a debilitating condition characterized by persistent fatigue and a range of complex accompanying symptoms. While curative treatments remain limited, Tuina (a traditional manual therapy in Chinese medicine) has shown potential in alleviating fatigue in pilot studies. However, high-quality evidence from large-scale, multicenter trials regarding its efficacy and long-term sustainability is lacking.

**Objective:**

This trial aims to evaluate the efficacy and safety of Tuina combined with usual care (UC) for CFS compared with UC alone.

**Methods:**

This prospective, multicenter, randomized, open-label, assessor-blinded, parallel-group clinical trial will be conducted at 6 hospitals in China. The study consists of 3 phases: a 1-week run-in period, an 8-week treatment period, and a 24-week follow-up period. A total of 230 eligible participants diagnosed with CFS (Centers for Disease Control and Prevention 1994 criteria) will be recruited and randomly allocated (1:1) to receive either Tuina therapy plus UC (intervention group) or UC alone (control group). The intervention group will undergo 24 sessions of a standardized Tuina protocol over 8 weeks (3 sessions/week). Follow-up assessments will be conducted at weeks 4, 8, 20, and 32. The primary outcome is the change in fatigue severity measured by the 11-item Chalder Fatigue Questionnaire from baseline to week 8. Secondary outcomes include clinical response rate, sleep quality, anxiety, depression, quality of life, adverse events, and clinical global impression. Efficacy analysis will be performed using a mixed-effects model for repeated measures in the modified intention-to-treat population. Secondary outcomes will be analyzed as exploratory supportive end points using appropriate parametric or nonparametric tests based on data distribution, with week 8 defined as the main secondary time point and later follow-up visits interpreted as durability contrasts. Missing outcome data will be handled primarily through likelihood-based mixed-model estimation under a missing-at-random assumption, with multiple imputation and per-protocol analyses used as sensitivity analyses.

**Results:**

Recruitment for this trial will start in March 2026 and is expected to be completed by March 2027. The results of this study are expected to provide reliable evidence regarding the role of Tuina in the management of CFS.

**Conclusions:**

This multicenter randomized controlled trial will evaluate the effectiveness, long-term sustainability, and safety of Tuina therapy plus UC for improving fatigue, sleep, mood, and quality of life in CFS or ME. If proven beneficial, Tuina may serve as a scalable nonpharmacological option to support clinical decision-making and standardized implementation.

## Introduction

Chronic fatigue syndrome (CFS), also known as myalgic encephalomyelitis (ME), represents a significant global health challenge, characterized by persistent or relapsing fatigue and a range of complex, medically unexplained accompanying symptoms [1,2]. The global prevalence of CFS is estimated to be between 0.4% and 2.6%, and incidence rates may be rising due to modern lifestyle stressors and postviral sequelae (eg, long COVID) [2,3]. Individuals with CFS or ME may experience difficulties in working, education, and mobility. In severe cases, their condition may confine them to bed and render them dependent on care [4]. Given its unclear etiology, curative treatments remain elusive [1,5-7].

The management of CFS has traditionally emphasized graded exercise therapy (GET) and cognitive behavioral therapy (CBT) [5-7]. However, the safety of these interventions has been questioned, leading the 2021 National Institute for Health and Care Excellence (NICE) guidelines to withdraw GET due to concerns over symptom exacerbation, and downgrade CBT to a supportive role [8,9]. These limitations highlight a critical unmet need for nonpharmacological interventions that are both effective and well tolerated.

Tuina is a traditional Chinese medicine external therapy based on the fundamental theories of traditional Chinese medicine. Through specific manipulations such as pressing, kneading, and pushing on the meridians and acupoints, Tuina aims to promote the flow of Qi and blood, thereby restoring physiological balance [10]. Emerging evidence suggests that Tuina is a promising approach for alleviating CFS or ME symptoms, showing potential benefits in reducing fatigue, improving sleep quality, and alleviating anxiety and depression [11-13]. In addition, a recent systematic review and meta-analysis of massage therapy for CFS reported improvements in fatigue symptoms and few adverse reactions, although the included studies were heterogeneous and generally limited in methodological rigor [14].

However, despite these promising findings, the current evidence base remains limited. Most existing studies are characterized by small sample sizes, single-center designs, and short-term follow-ups, which may restrict the generalizability of the results [15,16]. Furthermore, the lack of standardized intervention protocols across different clinical settings poses a challenge to evaluating the consistency of Tuina’s therapeutic effects. Consequently, high-quality evidence from large-scale, multicenter trials with rigorous methodology is urgently needed to substantiate the efficacy of Tuina in clinical management for CFS or ME.

## Methods

### Objective

The primary objective is to evaluate the efficacy of an 8-week standardized Tuina therapy plus usual care (UC) compared to UC alone in reducing fatigue severity in patients with CFS. Secondary objectives include assessing effects on the clinical response rate, sleep quality, mental health, quality of life, and clinical global impression, as well as evaluating the safety of the intervention and the long-term sustainability of therapeutic effects up to 24 weeks after treatment.

### Trial Design

This study uses a prospective, multicenter, randomized, open-label, assessor-blinded, parallel-group design. The trial will be conducted at 6 clinical centers across China, with Shuguang Hospital affiliated to Shanghai University of Traditional Chinese Medicine serving as the leading center. The protocol has been developed in accordance with the SPIRIT (Standard Protocol Items: Recommendations for Interventional Trials) 2025 statement (Checklist 1) [17]. Table 1 outlines the major time points of this trial.

**Table 1.** Schedule of enrollment, interventions, and assessments^a^.

Study period	Run-in	Allocation	Treatment	End of treatment	Follow-up	Follow-up	Other
Timepoint	Week −1 to 0	Week 0	Week 4	Week 8	Week 20	Week 32	Early withdrawal
Enrolment							
Informed consent	✓						
Eligibility screening	✓	✓					
Demographics	✓						
Medical history	✓						
Randomization		✓					
Interventions							
Tuina plus UC^b^			✓	✓			✓
UC			✓	✓			✓
Assessments							
CFQ-11^c^		✓	✓	✓	✓	✓	✓
SF-36 PF^d^	✓	✓	✓	✓	✓	✓	✓
SF-36 BP^e^		✓	✓	✓	✓	✓	✓
HADS^f^	✓	✓	✓	✓	✓	✓	✓
PSQI^g^		✓	✓	✓	✓	✓	✓
EQ-5D-3L		✓	✓	✓	✓	✓	✓
CGI^h^				✓			✓
Safety and other data							
Vital signs		✓	✓	✓			✓
Physical examination		✓	✓	✓			✓
Adverse events		✓	✓	✓	✓	✓	✓
Concomitant medications and therapies		✓	✓	✓	✓	✓	✓
UC log and consultation record		✓	✓	✓	✓	✓	✓
Adherence check			✓	✓			✓

a The “✓” symbol indicates that the item is scheduled at that time point.

b UC: usual care.

c CFQ-11: Chalder Fatigue Questionnaire.

d SF-36 PF: Short-Form 36 Physical Function Subscale.

e SF-36 BP: SF-36 Bodily Pain Subscale.

f HADS: Hospital Anxiety and Depression Scale.

g PSQI: Pittsburgh Sleep Quality Index.

h CGI: clinical global impression.

The study timeline is divided into three phases:

1. Run-in phase (day −7 to day 0): A 1-week screening period designed to confirm eligibility, ensure participant compliance, and collect stable baseline data (specifically depression levels and physical function) to serve as stratification factors for the subsequent randomization.
2. Treatment phase (week 1 to week 8): Participants will receive the assigned intervention (Tuina plus UC or UC alone) for 8 consecutive weeks.
3. Follow-up phase (week 9 to week 32): A 24-week posttreatment follow-up period to evaluate the long-term sustainability of the therapeutic effects.

### Patients and Recruitment

In this multicenter trial, patients with CFS will be recruited from outpatient clinics at 6 participating hospitals through advertisements on posters, WeChat, and hospital websites. Specialized physicians will confirm the diagnoses based on the 1994 Centers for Disease Control and Prevention (CDC) criteria, using comprehensive medical history taking, physical examinations, and necessary laboratory tests to exclude other organic or psychiatric causes of fatigue. To promote recruitment and retention, the treatment and relevant clinical assessments involved in the study will be offered free of charge to eligible candidates. Written informed consent will be obtained from all participants before the screening. Eligibility criteria are presented in a 2-column format in Textbox 1.

Textbox 1. **Eligibility criteria.**

**Inclusion criteria**

1. Diagnosis of chronic fatigue syndrome or myalgic encephalomyelitis according to the 1994 Centers for Disease Control and Prevention criteria [18], which includes the following:
  - (1) persistent or recurrent fatigue lasting more than 6 months; (2) other organic diseases causing chronic fatigue must be excluded based on medical history, physical examination, or laboratory findings.
  - The concurrent occurrence of at least 4 of the following 8 symptoms: (1) self-reported impairment in short-term memory or concentration; (2) sore throat; (3) tender cervical or axillary lymph nodes; (4) muscle pain; (5) multijoint pain without joint swelling or redness; (6) headaches of a new type, pattern, or severity; (7) unrefreshing sleep; and (8) post-exertional malaise lasting more than 24 hours.
2. Age between 18 and 65 years (inclusive), with no gender restrictions.
3. Willingness to participate in the study and capability to provide written informed consent.

**Exclusion criteria**

1. Secondary fatigue caused by other identifiable conditions, including infectious diseases, autoimmune disorders, malignancies, hypothyroidism, diabetes mellitus (and its complications), or drug-induced fatigue.
2. Severe organic or unstable chronic diseases involving the cardiovascular, cerebrovascular, hepatic, renal, respiratory, or hematological systems.
3. Long-term dependency on glucocorticoids with an inability to discontinue usage.
4. High risk of bleeding, such as thrombocytopenia or coagulation disorders.
5. Initiation of new treatments (pharmacological or non-pharmacological) for CFS or related symptoms within 14 days prior to enrollment.
6. Skin conditions at the treatment sites or other physical conditions assessed by the investigator as unsuitable for Tuina therapy.
7. Diagnosis of anorexia or anorexia nervosa.
8. Severe psychiatric comorbidities, including schizophrenia, bipolar disorder, or current suicidal ideation.
9. Severe obesity, defined as a BMI >45 kg/m².
10. Pregnant or lactating women.
11. Participation in another clinical trial within the past month or concurrent participation in other trials.
12. Any other condition that, in the opinion of the investigator, renders the participant unsuitable for the study.

### Randomization and Blinding

Eligible participants will be randomized in a 1:1 ratio to either the intervention group (Tuina plus UC) or the control group (UC) via a central web-based randomization system (Interactive Web Response System [IWRS]). To ensure balance between groups across the 6 centers, the minimization method will be used. Stratification factors, based on baseline data collected at the end of the run-in phase, include:

1. Depression severity: Hospital Anxiety and Depression Scale (HADS)-Depression score (≤10 vs ≥11).
2. Physical function: 36-item Short Form Health Survey (SF-36) Physical Functioning subscale score (≤40 vs ≥45).

### Allocation Concealment

The randomization sequence is generated by the IWRS and remains concealed from all investigators, therapists, and participants until the moment of assignment. Researchers will obtain the group assignment only after confirming eligibility and completing baseline assessments.

### Blinding

Because Tuina is a manual therapy, neither participants nor therapists can be blinded to group allocation. This open-label design may introduce performance and expectation bias. Moreover, most efficacy outcomes, including the 11-item Chalder Fatigue Questionnaire (CFQ-11), Pittsburgh Sleep Quality Index (PSQI), HADS, SF-36, and EQ-5D-3L, are patient-reported; therefore, assessor blinding provides limited protection against bias for these self-reported outcomes. This limitation is explicitly acknowledged and will be mitigated by standardized participant instructions, equal assessment schedules, electronic or paper questionnaire completion before clinical discussion, and separation of treatment providers from outcome assessors.

Outcome assessors responsible for collecting data will be blinded to group allocation and will not participate in treatment. The CGI will be assessed by clinicians who are blinded to allocation whenever feasible. Independent statisticians will analyze data using masked group codes until the primary analysis is finalized. Participants will be instructed not to disclose treatment details to assessors during follow-up visits.

### Interventions

#### Control Group (UC)

Based on current guidance [1], participants in the control group will receive UC delivered by physicians experienced in CFS management. UC will include a baseline professional evaluation and structured education covering the nature of CFS, pacing and energy conservation, sleep hygiene, balanced diet, avoidance of overexertion and symptom-contingent activity escalation, and advice to avoid initiating Tuina, acupuncture, massage, or other traditional Chinese manual or physical therapies during the trial. This UC framework will be applied at all centers using the same written guidance document.

Symptom-directed treatment may be provided when clinically indicated for insomnia, pain, anxiety, depression, or other relevant symptoms. This may include prescription or nonprescription medications and individualized medical advice. Initiation or dose changes of medications and additional consultations will be permitted when clinically necessary, but the reason, drug name, dose, frequency, duration, and prescribing physician will be recorded in the electronic case report form. UC exposure will be recorded for both groups at each assessment visit using a structured UC log that captures medical advice, consultations, medications, self-management recommendations, and nonstudy therapies. Differences in UC exposure between the groups will be summarized, and sensitivity analyses may adjust for major concomitant medications or prohibited cointerventions if imbalances occur.

#### Intervention (Tuina Plus UC)

Participants in the Tuina group will receive the same UC framework in addition to the Tuina protocol.

### Practitioner Qualification and Training

Tuina will be delivered by licensed practitioners with at least 5 years of clinical experience. Before trial initiation, all practitioners will complete centralized training consisting of 4 two-hour sessions within 1 week. Training will include theoretical instruction, video demonstration, supervised hands-on practice, calibration of manipulation frequency and force, and review of the trial standard operating procedure (SOP). Practitioners must pass a competency assessment before treating trial participants.

### Standardized Tuina Protocol

#### Overview

**Figure 1.**
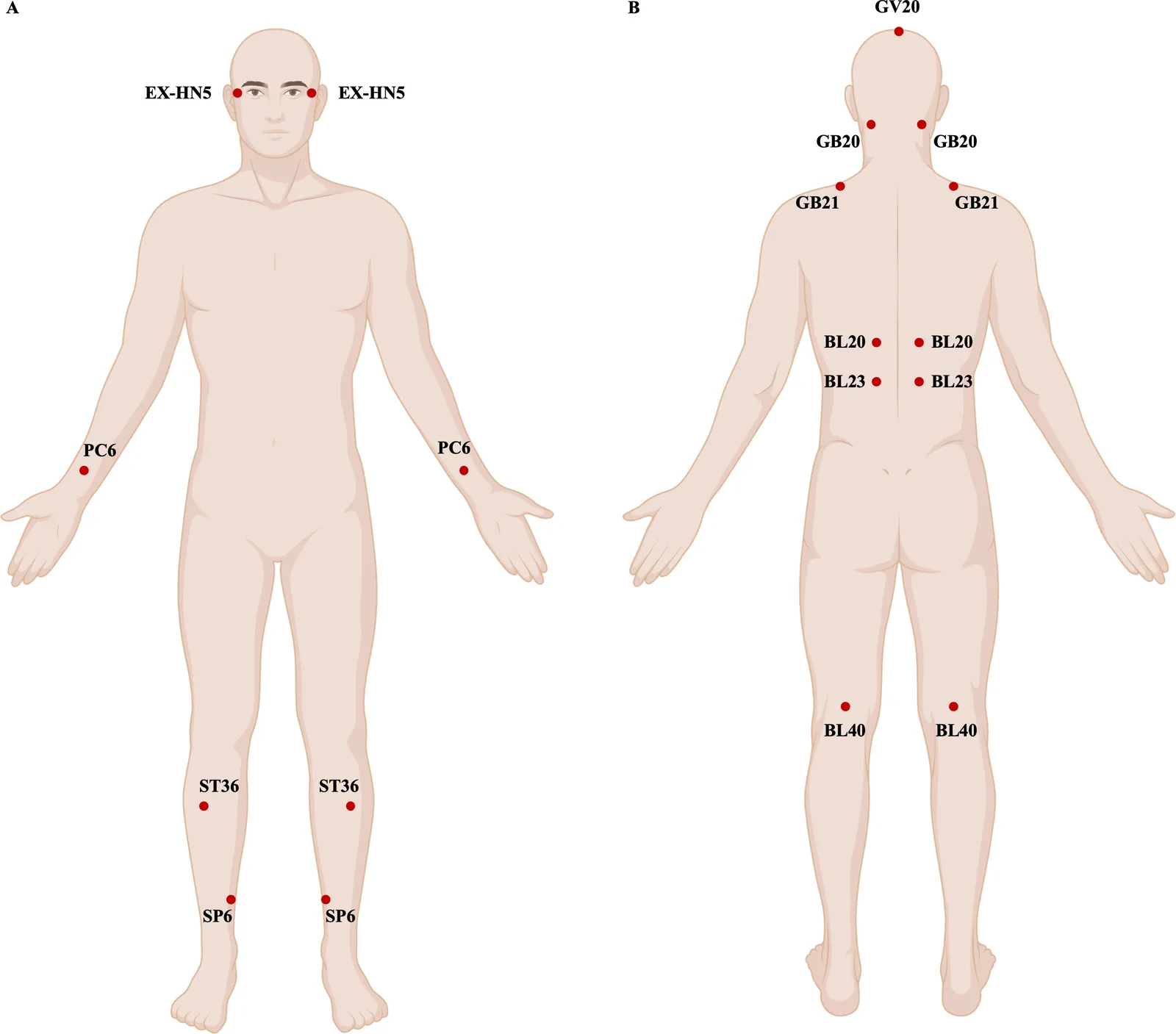
Locations of the acupoints used in the Tuina protocol. (A) Anterior view showing the locations of Taiyang (EX-HN5) on the temples, Neiguan (PC6) on the forearm, Zusanli (ST36) on the lower leg, and Sanyinjiao (SP6) on the medial aspect of the leg. (B) Posterior view showing the locations of Baihui (GV20) on the vertex of the head, Fengchi (GB20) on the nape, Jianjing (GB21) on the shoulder, Pishu (BL20), Shenshu (BL23), and Weizhong (BL40) along the Bladder Meridian on the back and posterior leg. Image created with bioRender [19].

An SOP will be strictly followed. The therapy will be administered 3 times per week for 8 consecutive weeks, totaling 24 sessions. Each session will last approximately 30 minutes. The protocol aims to regulate the spleen, liver, and kidney meridians by targeting specific regions and acupoints. The primary technique is pressing-kneading (An-Rou), supplemented by rolling (Gun), and pushing (Tui). The anatomical locations of the selected acupoints are shown in Figure 1.

#### Head and Face (Supine Position, 6 Minutes)

Manipulation of the head and face in the supine position for 6 minutes.

- Acupoints: *Taiyang* (EX-HN5), *Baihui* (GV20), *Fengchi* (GB20).
- Manipulation: The practitioner uses the thumb to apply pressing (An) and kneading (Rou) manipulations on these acupoints to tranquilize the mind. Each point is stimulated for approximately 2 minutes.

#### Upper Limbs (Supine Position, 2 Minutes)

Manipulation of the upper limbs in the supine position for 2 minutes.

- Acupoints: *Neiguan* (PC6) bilaterally.
- Manipulation: Pressing-kneading is applied to *Neiguan* to regulate the heart and spirit. The practitioner also performs pushing (Tui) along the pericardium meridian on the medial side of the forearm.

#### Lower Limbs (Supine Position, 4 Minutes)

Manipulation of the lower limbs in the supine position for 4 minutes.

- Acupoints: *Zusanli* (ST36) and *Sanyinjiao* (SP6) bilaterally.
- Manipulation: Pressing-kneading is performed on these points to strengthen the spleen and stomach, nourish *Yin*, and tonify *Qi*.

#### Back and Posterior Lower Limbs (Prone Position, 18 Minutes)

Manipulation of the back and posterior lower limbs in the prone position for 18 minutes.

- Regions: The back, hips, and posterior aspect of the legs (along the Bladder Meridian).
- Acupoints: *Jianjing* (GB21), *Pishu* (BL20), S*henshu* (BL23), *Weizhong* (BL40), and *Ashi* points (tender points).
- Manipulation:
  1. Rolling (Gun): Applied along the Bladder Meridian on both sides of the spine (from the neck down to the lumbar region) to relax the paraspinal muscles.
  2. Pressing-kneading: Specifically applied to the focus acupoints to regulate visceral function.
  3. Pushing (Tui): Performed downward along the back and the posterior aspect of the legs to the heels to promote the flow of Qi and blood.

#### Treatment Regimen

##### Manipulation Parameters

Pressing-kneading will be maintained at approximately 120 repetitions per minute. During practitioner training and periodic quality checks, stimulation force will be calibrated using a pressure-measuring device or electronic scale, targeting an average force of 3.0 (SD 0.5) kg for pressing-kneading. Rolling will be performed rhythmically at approximately 120 to 160 oscillations per minute, and pushing will be performed at approximately 20 to 30 strokes per minute. Intensity will be adjusted to patient tolerance and reduced if discomfort exceeds 3 on a 0 to 10 numeric rating scale.

##### Intervention Fidelity

Practitioners will complete a treatment log after every session documenting date, duration, manipulation techniques, approximate force and frequency, deviations from the SOP, and adverse events. The coordinating center will review treatment logs regularly, observe or video-audit a random sample of sessions, and provide feedback or retraining if protocol deviations are identified. Participants who miss sessions will be contacted to encourage adherence, and missed sessions may be rescheduled within the 8-week treatment window when feasible.

### Outcome Measurements

#### Primary Outcome

The primary outcome is the change in fatigue severity from baseline to the end of the intervention (week 8). Fatigue severity will be measured using the CFQ-11 [20]. The CFQ-11 is a validated self-report instrument widely used to evaluate the degree of fatigue in clinical and epidemiological studies [21,22]. It consists of 11 items divided into 2 dimensions: physical fatigue (items 1‐7) and mental fatigue (items 8‐11). Each item is rated on a 4-point Likert scale (0=“less than usual,” 1=“no more than usual,” 2=“more than usual,” and 3=“much more than usual”). The total score is calculated by summing the scores of all items, ranging from 0 to 33. Higher scores indicate a greater severity of fatigue.

#### Secondary Outcomes

##### Clinical Response Rate

###### Overview

To evaluate the clinical significance of the treatment effect, the clinical response rate will be calculated at week 8. A responder is defined as a participant who achieves a reduction of ≥3 points in the CFQ-11 total score compared to baseline. This prespecified threshold was chosen as a conservative clinically meaningful improvement criterion informed by published work on minimal clinically important difference (MCID) for fatigue patient-reported outcome measures [23].

###### Sleep Quality

Sleep quality over the previous month will be assessed using the PSQI [24]. This questionnaire comprises 19 self-rated items that are grouped into 7 component scores: sleep quality, sleep latency, sleep duration, sleep efficiency, sleep disturbances, use of sleeping medication, and daytime dysfunction. Each component is scored from 0 to 3. The sum of these 7 component scores yields a global PSQI score ranging from 0 to 21, with higher scores indicating poorer sleep quality.

### Mental Health Status

Symptoms of anxiety and depression will be evaluated using the HADS. This scale consists of 14 items, divided into two subscales: Anxiety (HADS-A, 7 items) and Depression (HADS-D, 7 items). Each item is scored on a scale of 0 to 3. The scores for each subscale range from 0 to 21, with higher scores indicating more severe symptoms of anxiety or depression.

### Quality of Life

Health-related quality of life will be assessed using the SF-36 [25]. This instrument measures 8 domains of health status: physical functioning (PF), role limitations due to physical health (RP), bodily pain (BP), general health (GH), vitality (VT), social functioning (SF), role limitations due to emotional problems (RE), and mental health (MH). For each domain, item scores are coded, summed, and transformed onto a scale from 0 (worst possible health state) to 100 (best possible health state). This study will specifically focus on the PF and BP subscales to assess functional improvement. For the PF subscale used as a stratification factor, the 10 PF items will be summed as a raw score ranging from 10 to 30 and transformed using the formula PF = (raw score − 10)/20 × 100; therefore, possible PF scores occur in 5-point increments.

Additionally, the EQ-5D-3L will be used to evaluate health utility. It covers 5 dimensions: mobility, self-care, usual activities, pain or discomfort, and anxiety or depression. Each dimension has 3 levels: no problems, some problems, and extreme problems. The responses are converted into a single utility index using the Chinese population-based value set [26]. The scale also includes a EuroQol Visual Analogue Scale (EQ-VAS), where participants rate their overall health on a vertical scale from 0 (worst imaginable health) to 100 (best imaginable health).

### Global Impression

The Clinical Global Impression (CGI) scale will be used to assess the clinician’s global view of the patient’s illness and improvement [27]. It includes two subscales: Severity of Illness (CGI-S), rated on a 7-point scale from 1 (normal, not at all ill) to 7 (among the most extremely ill patients), and Global Improvement (CGI-I), rated from 1 (very much improved) to 7 (very much worse).

### Safety and Adverse Event Reporting

Adverse events (AEs) in this trial are defined as any untoward medical occurrence in a participant, regardless of its causal relationship with the treatment. Serious adverse events (SAEs) refer to events that result in death, are life-threatening, require hospitalization or prolonged hospitalization, or cause significant disability. AEs will be comprehensively monitored and recorded for both the intervention and control groups throughout the study. Specifically, monitoring will focus on intervention-related events such as subcutaneous hemorrhage (bruising), persistent local pain, skin abrasion, or fainting in the Tuina group, while simultaneously assessing potential side effects of concomitant medications or symptom fluctuation in the UC group. The number and type of AEs will be calculated, and patients will receive appropriate intervention if necessary. Any SAEs must be reported to the principal investigator and the Ethics Committee within 24 hours, and affected participants may be withdrawn from the study to ensure safety.

### Statistical Analysis

The sample size was determined based on the primary outcome, namely the change in CFQ-11 total score from baseline to week 8. The variability estimate was derived from individual-level data from an investigator-initiated, single-center pilot study conducted before the current multicenter protocol was finalized. In that pilot study, the mean reduction in CFQ-11 total score was 10.29 (SD 6.02) in the Tuina group and 5.36 (SD 6.39) in the control group. Because the primary end point of the present trial is change from baseline, the SDs of the baseline-to-posttreatment change scores were used for sample size planning. These values differ from the posttreatment group-level SDs reported in the pilot study table, which described the distribution of CFQ-11 scores at the posttreatment visit rather than the distribution of change scores. The pooled SD was calculated using the following formula:


$${SD}_{pooled}=\sqrt{\frac{(n_{1}-1)\times {{SD}_{1}}^{2}+(n_{2}-1)\times {{SD}_{2}}^{2}}{n_{1}+n_{2}-2}}$$

With n_1_=n_2_=55, SD_1_=6.02, and SD_2_=6.39, the pooled SD was as follows:


$${SD}_{pooled}=\sqrt{\frac{(55-1)\times 6.02^{2}+(55-1)\times 6.39^{2}}{55+55-2}}=6.21$$

The expected between-group difference was set at 3.0 points, corresponding to the prespecified clinically useful improvement threshold for CFQ-11 [23]. This conservative choice was made because the pilot study was single-center and relatively small, and pilot-based treatment effect estimates may be unstable or overestimate the effect expected in a larger multicenter trial. Using the MCID as the target effect ensures that the trial is powered to detect a clinically meaningful difference. To detect a 3.0-point between-group difference with a pooled SD of 6.21, 90% power, and a 2-sided significance level of 0.05, 92 participants per group are required. Allowing for a 20% dropout rate, the target sample size is 115 participants per group, for a total of 230 participants. The calculation was performed using PASS software.

### Data Analysis Plan

#### Analysis Populations

The primary efficacy analysis will be based on the modified intention-to-treat (mITT) population, operationalized as the full analysis set (FAS). The FAS will include all randomized participants with valid baseline CFQ-11 data who either receive at least 1 component of the allocated study management after randomization or provide at least 1 postbaseline CFQ-11 assessment. Study management refers to Tuina and/or usual care in the intervention group and usual care in the control group. Participants in the FAS will be analyzed according to their randomized allocation, irrespective of adherence, treatment discontinuation, number of completed sessions, protocol deviations, or concomitant care.

Participants who do not initiate any allocated postrandomization study management but provide valid postbaseline CFQ-11 data will remain in the FAS and will be analyzed according to randomized allocation. Postrandomization exclusions from the FAS will be limited to participants who withdraw consent for use of all data, have no valid baseline CFQ-11 data, are found after randomization to have a major eligibility error, or withdraw immediately after randomization before any study management and provide no postbaseline primary outcome data. All such exclusions will be documented before database lock and summarized in the participant flow diagram.

The per-protocol set (PPS) will include participants who complete at least 80% of the assigned intervention requirements, have week 8 primary outcome data, and have no major protocol violations. The safety set (SS) will include all randomized participants who receive at least 1 component of the allocated intervention or usual care and will be analyzed according to treatment actually received.

#### Descriptive Statistics

Continuous data will be summarized as mean (SD) or median with IQR depending on data distribution, while categorical data will be presented as frequencies and percentages.

### Outcome Analysis

The primary outcome will be analyzed using MMRM based on the FAS. The model will include treatment group, visit, center, treatment-by-visit interaction, and baseline stratification factors (depression and physical function) as fixed effects, with the baseline CFQ-11 score a covariate. Within-participant correlations among repeated measurements will be modeled using an unstructured covariance matrix. If the model does not converge, alternative covariance structures such as first-order autoregressive or compound symmetry will be selected according to Akaike information criterion and convergence diagnostics. Restricted maximum likelihood estimation will be used. The primary contrast will be the adjusted between-group mean difference at week 8, with a 95% CI and 2-sided *P* value.

For secondary continuous outcomes, between-group differences in change from baseline will be compared at each scheduled postbaseline time point using 2-tailed Student *t* test when data are approximately normally distributed or the Wilcoxon rank-sum test when distributional assumptions are not met. This time point–specific approach is prespecified because secondary outcomes are supportive and exploratory end points, the trial is powered for the primary CFQ-11 outcome rather than for multiple longitudinal secondary scales, and the main clinical interpretation of secondary efficacy will focus on the end-of-treatment assessment. Week 4 will be used to describe early treatment response, week 8 will be defined as the main secondary efficacy time point, and weeks 20 and 32 will be used to assess durability and will be interpreted as supportive follow-up contrasts. For categorical secondary outcomes, the *χ*^2^ test or Fisher exact test will be used, as appropriate. The clinical response rate at week 8 will be compared between the groups using logistic regression adjusted for baseline CFQ-11 score, center, and baseline stratification factors. Ordinal CGI outcomes will be analyzed using proportional-odds models when the proportional-odds assumption is met; otherwise, nonparametric methods will be used. AE rates will be summarized descriptively and compared using the *χ*^2^ test or Fisher exact test when appropriate.

To address multiplicity among secondary efficacy outcomes, *P* values for the prespecified week 8 secondary efficacy outcomes will be adjusted using the Benjamini-Hochberg false discovery rate procedure at 5%. Week 4, week 20, and week 32 contrasts will not be included in this multiplicity family; these follow-up contrasts will be reported with effect estimates and 95% CIs and interpreted as exploratory supportive evidence of early response or durability rather than as confirmatory efficacy tests.

### Missing Data

All missing data will be documented with reasons whenever possible and summarized by group and visit. No last-observation-carried-forward approach will be used. The primary MMRM analysis handles missing repeated outcome data through likelihood-based estimation under the missing-at-random assumption, using all available observed data. Multiple imputation by chained equations will be performed as a sensitivity analysis for the primary outcome and key secondary continuous outcomes when postbaseline data are missing. The imputation model will include treatment group, center, baseline stratification factors, baseline outcome values, observed postbaseline outcomes, demographic variables, and relevant UC or concomitant-treatment variables. At least 50 imputed datasets will be generated, and estimates will be combined using Rubin rules [28-30]. For the clinical response rate, response status will be derived from imputed CFQ-11 values in the MI sensitivity analysis; an additional conservative sensitivity analysis will classify participants with missing week 8 CFQ-11 data as nonresponders. If overall missingness exceeds 20% or differs between groups by more than 10 percentage points, additional sensitivity analyses using delta-adjusted pattern-mixture methods will be considered to assess departures from the missing-at-random assumption. Additionally, the robustness of the primary outcome will be assessed using the PPS population as a sensitivity analysis.

All statistical analyses will be performed using SAS version 9.4 (SAS Institute). Statistical significance is defined as a 2-sided *P*<.05.

### Ethical Considerations

Ethical approval for this study was obtained from the Ethics Committee of Shuguang Hospital affiliated to Shanghai University of Traditional Chinese Medicine, China (approval number 2025-1860-200-02), and the study will be conducted in accordance with the Declaration of Helsinki. All participants will be fully informed of the study objectives, procedures, potential risks, and anticipated benefits, and written informed consent will be obtained prior to enrollment.

Participant confidentiality will be strictly maintained throughout the study. Access to identifiable data will be restricted to authorized members of the research team, and all data will be anonymized for analysis. Study findings will be disseminated through peer-reviewed publications, and no identifiable personal information or images will be included in any published materials. Data will be collected and managed using a secure, web-based electronic data capture (EDC) system. To ensure data integrity, the system incorporates automated logic checks, range validations, and a complete audit trail of all data modifications. All participant data will be deidentified using unique study identification numbers to protect confidentiality. Regular data backups will be performed, and access to the database will be strictly restricted to authorized personnel with individual passwords. Patients and the public were not formally involved in the design, conduct, reporting, or dissemination plans of this study.

## Results

Recruitment for this multicenter randomized controlled trial is scheduled to commence in March 2026. Eligible patients will be enrolled and randomly assigned to either the Tuina plus UC group or the UC group. The intervention period for each participant will last 8 weeks, followed by a 24-week follow-up period. Recruitment is expected to be completed by March 2027, and the intervention for the final participant is anticipated to conclude in May 2027. All follow-up assessments and data collection procedures are expected to be finalized by November 2027. Subsequently, data analysis and reporting of the primary and secondary outcomes are anticipated to be completed in early 2028.

## Discussion

### Principal Findings

This protocol describes a multicenter randomized trial designed to test the hypothesis that 8 weeks of standardized Tuina therapy plus UC will produce a clinically meaningful reduction in fatigue severity compared with UC alone among patients with CFS. We also hypothesize that Tuina will increase the clinical response rate, improve sleep, mood symptoms, physical function, bodily pain, and health-related quality of life, and show acceptable safety. The 24-week posttreatment follow-up will allow the assessment of whether any treatment benefits are sustained beyond the active intervention period.

### Comparison With Prior Work

CFS creates a substantial burden on individuals and health care systems. As highlighted by the 2021 NICE guidelines, standard nonpharmacological interventions, such as GET, are no longer recommended due to safety concerns, while CBT is primarily supportive rather than curative [1,8,9]. This underscores the need for additional nonpharmacological approaches that are acceptable to patients and unlikely to exacerbate postexertional symptoms. Previous studies suggest that Tuina and related manual therapies may improve fatigue and accompanying symptoms [11-14,31], but the evidence remains limited by small samples, single-center recruitment, heterogeneous intervention protocols, and short-term follow-up [15,16]. The present trial addresses these gaps by using a larger multicenter design, a standardized treatment manual, centralized randomization, prespecified statistical methods, and longer follow-up.

### Strengths and Limitations

The study has several strengths. First, to our knowledge, this is the first large-scale, multicenter randomized controlled trial to investigate the efficacy and safety of Tuina therapy for CFS. The study design incorporates several methodological strengths to ensure rigor. Second, building upon our preliminary single-center pilot work, we have optimized the intervention protocol. We extended the treatment duration from 4 weeks to 8 weeks and the session length to 30 minutes. This dose optimization aligns with the chronic nature of CFS and aims to induce more stable, long-term therapeutic effects. A major challenge in manual therapy trials is operator variability [32]. Third, we have implemented a strict quality control system, including a unified SOP, centralized practitioner training, systematic treatment logs, and regular fidelity monitoring. This ensures that the intervention is reproducible across different clinical centers and allows cointerventions and concomitant medications to be described and explored in sensitivity analyses. Fourth, this trial includes a 24-week posttreatment follow-up, enabling evaluation of durability.

Several limitations should be acknowledged. First, because Tuina is a manual therapy, participant and therapist blinding is infeasible [33,34]. This open-label design may introduce performance or expectation bias. To mitigate this, we have implemented a strict assessor-blinded design, where outcome evaluators and statisticians are unaware of group allocations. Second, the primary outcome (CFQ-11) and key secondary outcomes are patient-reported measures, which are subjective. However, these scales are validated and widely accepted in CFS research, and we have included the CGI to provide a clinician’s perspective. Third, the study population is limited to patients meeting the CDC 1994 criteria; future research may need to explore efficacy across different diagnostic definitions (eg, Institute of Medicine or NICE criteria) to enhance generalizability.

### Future Directions and Dissemination

If Tuina is shown to be effective and safe, it could serve as an adjunctive nonpharmacological option for CFS management. The standardized protocol may support practitioner training and facilitate implementation in clinical settings. Future research should examine pragmatic delivery models, cost-effectiveness, objective activity or physiological outcomes, mechanisms of action, and effectiveness across alternative CFS/ME diagnostic criteria. Trial findings will be disseminated through peer-reviewed publications, conference presentations, trial registry updates, and sharing of the standardized Tuina protocol where appropriate.

### Conclusions

This multicenter randomized controlled trial will evaluate the efficacy, durability, and safety of Tuina therapy plus UC for patients with CFS. The findings are expected to provide clinically relevant evidence for whether Tuina can be integrated into CFS management as a scalable nonpharmacological intervention.

## Supplementary material

10.2196/94022 Checklist 1 SPIRIT checklist.

## Abbreviations

AE: adverse event
BP: Bodily Pain (SF-36 subscale)
CBT: cognitive behavioral therapy
CDC: Centers for Disease Control and Prevention
CFQ-11: 11-item Chalder Fatigue Questionnaire
CFS: chronic fatigue syndrome
CGI: Clinical Global Impression
CGI-I: Clinical Global Impression—Improvement
CGI-S: Clinical Global Impression—Severity
EQ-VAS: EuroQol Visual Analogue Scale
FAS: full analysis set
GET: graded exercise therapy
GH: General Health (SF-36 subscale)
HADS: Hospital Anxiety and Depression Scale
IWRS: interactive web response system
MCID: minimal clinically important difference
MH: Mental Health (SF-36 subscale)
MI: multiple imputation
mITT: modified intention-to-treat
MMRM: mixed-effects model for repeated measures
NICE: National Institute for Health and Care Excellence
PF: Physical Functioning (SF-36 subscale)
PPS: per-protocol set
PSQI: Pittsburgh Sleep Quality Index
RCT: randomized controlled trial
SAE: serious adverse event
SF: Social Functioning (SF-36 subscale)
SF-36: 36-item Short Form Health Survey
SOP: standard operating procedure
SPIRIT: Standard Protocol Items: Recommendations for Interventional Trials
SS: safety set
UC: usual care
VT: vitality (SF-36 subscale)
